# Psychological Adjustment to Disease and Treatment: Commentary on Dekker (2024)

**DOI:** 10.1002/smi.70190

**Published:** 2026-06-17

**Authors:** Natasha Seaton, Madison Milne‐Ives, Hannah Proudfoot, Christina Joanne Pearce

**Affiliations:** ^1^ Department of Psychology King's College London London UK

Dekker's recent paper ‘Psychological adjustment to disease and treatment: A general model’ aims to integrate models of psychological adjustment to disease (Dekker 2024). Diagnosis and experience of diseases can trigger significant changes in a person's daily life and self‐perception (Dekker 2024; Carroll et al. 2022). Dekker's aim of synthesizing key factors about how people adjust to living with their disease is important. The simplified model proposed provides a good overview of the types of factors to consider and has great potential value for education regarding the process of adjustment. The following discussion focuses on two key aspects: the limitations of over‐simplicity in the model—in terms of relationships between the factors and the choice of outcome—and methodological concerns. We conclude with a summary of suggestions that we believe would increase the value and applicability of this model.

Dekker argues that having a simple, high‐level model adds value because it provides a comprehensive summary of relevant factors as a starting place for further research and theory comparison (Dekker 2024). While this argument has merit, the model's simplicity also raises challenges. As presented, the model functions more as a taxonomy than as a theory of adjustment. Although similarities and differences between the synthesized models are outlined (Dekker 2024), the framework does not specify how cognitive, emotional, behavioral, and physiological responses influence each other, nor does it articulate underlying mechanisms through which such interactions occur. This is despite substantial theory (e.g., predictive processing, embodied cognition, the theory of constructed emotion, and stress‐coping frameworks) and evidence demonstrating the dynamic interconnectedness of physical and mental processes (Koban et al. 2021). These perspectives are highly relevant for understanding psychological adjustment to disease.

To illustrate, we shall consider how Dekker's model might incorporate these theoretical approaches. The predictive processing framework posits that our brains are continuously generating expectations about bodily states and comparing them with incoming sensory inputs to update beliefs and guide future predictions (Putica and Agathos 2024). Perception is an active process, emerging from an integration of top‐down predictions and bottom‐up sensory data. As such, perception is influenced by a person's prior experiences. In the illness context, a prior negative symptom experience results in a prediction that may outweigh a neutral sensory input (Putica and Agathos 2024). Moreover, the relative weighting of these two systems is influenced by psychological and emotional context. There is evidence that negative affect leads to an over‐reliance on prior experiences and reduced interoceptive accuracy (Van den Bergh et al. 2017). This not only contributes to a confirmation bias where ambiguous cues are labeled as signs of disease activity, but additionally will limit corrective updating of priors, leading to symptom perception being shaped more by expectation than by objective damage signaled by sensory afferents (Barsky and Silbersweig 2023). Stress‐coping frameworks similarly posit that physiological stress response systems are partially contingent on subjective appraisal and emotional experience (Lazarus and Folkman 1984). Constructionist approaches to emotion propose that physiological activation can precede and exacerbate conscious emotion (Barrett 2017; Barrett et al. 2007). Taken together, these perspectives suggest that emotional, cognitive, and physiological responses are not parallel systems but mutually influential inferential processes, reflecting the brain's ongoing attempts to maintain stability and allostasis (Barrett 2017). To advance theory, models of psychological adjustment should incorporate mechanistic accounts—specifying how, why and when mental and physical systems interact, beyond simply presenting them as coexisting components.

The inclusion of ‘health’ as the outcome of the adjustment process is another aspect that we feel has been over‐simplified. While Dekker acknowledges that ‘health’ incorporates several domains, including ‘(i) disorder or disease, (ii) somatic and mental functions and structures, (iii) activities and participation, (iv) social and environmental factors, and (v) personal factors’ (Dekker 2024; Dekker et al. 2023), it is reductionist to interpret the adjustment process as determining ‘good’ or ‘poor’ health. Adaptation processes are inherently dynamic and temporal, but the visual representation implies a static, linear process (although its cyclicality is described in text). In the case of many long‐term conditions (LTCs), one's health interacts with a continuous adjustment process, as symptoms can change as the disease progresses. Therefore, the individual may experience ‘poor’ health (in terms of disease, functions, and activities), but they may be adjusting well to symptoms and illness stressors. The outcome of adjustment may be better framed as acceptance and psychological flexibility, rather than as restoration to ‘good’ health (McCracken 2024; Meek et al. 2022). This would depict a well‐adjusted person as living life in line with their goals and values, in the face of an LTC.

The choice of outcomes has practical and clinical implications. There is evidence that psychological flexibility—‘the ability to adapt to constantly changing environments’ (Wang et al. 2025)—can be negatively affected by traumatic experiences, such as an LTC diagnosis or a particularly severe experience with a disease symptom. Higher psychological flexibility has been associated with improve functioning and health outcomes (McCracken 2024), while lower psychological flexibility may increase vulnerability to suicidal ideation (Crasta et al. 2020; McCracken et al. 2018; De Berardis et al. 2020). While Dekker's concept of health may have included such psychological flexibility, this was not sufficiently clear (Dekker 2024), and a focus only on ‘health’ more generally may overlook this critical outcome. Arguably, adjustment and health cannot be disentangled or seen as cause and effect. Instead, the model could position health as an experience that occurs concurrently, and interacts with, the adjustment process.

Dekker positions the article as a commentary and is transparent that no data were analyzed, so no empirical testing is expected; however, this limits the model's utility. There is no discernible methodological justification for the selection of models. The paper excludes more recent models and commentaries that have explicitly attempted to advance the field. Notably, the article has omitted the 2022 Transdiagnostic Model of Adjustment to LTCs (TMA‐LTC) (Carroll et al. 2022), which builds on the included 2013 working model of adjustment to chronic illness (Moss‐Morris 2013). Dekker's critique states that all models, except the allostatic load model, exclude physiological responses; however, the TMA‐LTC incorporates biological mechanisms and consequences of adjustment. While systematic searches are time consuming, progress in the field depends on a rigorous and transparent approach to model inclusion to ensure recognition of recent developments.

Relatedly, the process of ‘abstracting from and integrating the four previous models’ (Dekker 2024) is asserted rather than demonstrated. No procedural detail is provided to indicate how theoretical components were identified or synthesized. A formal meta‐synthesis or conceptual mapping would have strengthened the methodological rigor and reproducibility. This issue may reflect the paper's unclear theoretical positioning—clarifying the model's intended use as a meta‐framework (to *describe* integration), or a process theory (to enable *prediction* or *explanation*) would guide methodology and clarify how it can most appropriately be applied in practice. Consequently, we feel the claim of the model's heuristic function is somewhat overstated. While heuristic models prioritize practical insight over theoretical completeness, Dekker's model does not specify which relationships warrant additional empirical testing, and how its constructs could be operationalized, which would be valuable for its use as a practical and pragmatic tool. However, we support its use for educational purposes, as it provides a helpful starting point for triangulating different theories.

We appreciate Dekker's aim of synthesizing various theories around psychological adjustment to disease, as this is a necessary process for advancing the field and ensuring the comprehensiveness and practical utility of our models. In this commentary, we have described key areas where the model could be improved, specifically: engaging with broader contemporary theoretical developments to enable a more precise account of how the different factors included in the model interact and clarifying the theoretical positioning and intended scope of the general model. For example, as a meta‐framework, it would be improved through explicit methodology for identifying, selecting and integrating component theories and constructs, ideally through a structured conceptual synthesis. Alternatively, as a process theory, the model must precisely define the cognitive, emotional, behavioral, and physiological responses and the relationships between them (including temporal dynamics and reciprocity). Operational definitions and examples of measurable constructs would facilitate empirical application and support theory‐driven testing and refinement of the model. These improvements would have practical and clinical benefits as it would allow for the development of more process‐based interventions that could be applied flexibly across different clinical contexts, illness trajectories and individual needs.

## Funding

M.M.I and H.P. are funded as part of a larger research program, REFUEL‐MS, led by Professor Rona Moss‐Morris, based in the Health Psychology Section, Institute of Psychiatry, Psychology & Neuroscience, at King's College London and delivered through the NIHR Maudsley Biomedical Research Center. The program is co‐funded by the National Institute for Health and Care Research (NIHR)'s Program Grant for Applied Research (PGfAR) funding stream (NIHR unique award identifier: NIHR203290) and the MS Society (Award reference: 151). The NIHR Maudsley Biomedical Research Center fully funds C.P. and partially funds N.S. The views expressed are those of the authors and not necessarily those of the NIHR, the Department of Health and Social Care, or the MS Society.

## Conflicts of Interest

One of the models examined in Dekker's (2024) paper was developed by the lead of the authors' research group (Professor Rona Moss‐Morris). None of the authors were involved in its development, but it is disclosed as a potential competing interest. The authors declare that there are no other conflicts of interest.

## Data Availability

Data sharing not applicable to this article as no datasets were generated or analysed during the current study.
